# ﻿Two new species of the order Monhysterida (Nematoda) from the sea of China

**DOI:** 10.3897/zookeys.1193.110188

**Published:** 2024-02-29

**Authors:** Ting Li, Yong Huang, Mian Huang

**Affiliations:** 1 College of Life Sciences, Liaocheng University, Liaocheng 252059, China Liaocheng University Liaocheng China

**Keywords:** Biodiversity, epiphytic, free-living marine nematode, *Halomonhysterazhangi* sp. nov., *Stylotheristusflagellicaudatus* sp. nov., taxonomy

## Abstract

Two new marine nematode species belonging to the order Monhysterida are described from the sea of China. *Halomonhysterazhangi***sp. nov.** is characterized by its relatively small body size; short anterior sensory setae; small, circular amphidial fovea located at the level of buccal cavity base; funnel-shaped buccal cavity; an excretory-secretory system with a large ventral gland and opening as a very narrow canal just posterior to the level of cephalic setae; slightly curved slender spicules with cephalated proximal end and tapered distal end; rod-like gubernaculum without apophysis; two papilliform precloacal supplements just in front of the cloaca; conico-cylindrical tail with two ventral papillae, each with a seta; and distance between the vulva and anus slightly longer than the tail length. This is the first new species of epiphytic nematode reported in China. The second new species, *Stylotheristusflagellicaudatus***sp. nov.**, has a relatively shorter body and longer tail; striated cuticle; The anterior sensilla arranged in two circles, the first circle consisting of six inner labial setiform papillae (3–4 µm) and the second circle consisting of 16 long setae (12–19 µm); a transversely oval amphideal fovea; a well-developed muscle around the funnel-shaped buccal cavity; short spicules and a gubernaculum composed of a single piece; and precloacal supplements absent. An updated key to all species of *Halomonhystera* and pictorial key to all species of *Stylotheristus* are also given.

## ﻿Introduction

To investigate the diversity of epiphytic nematodes growing in seaweed in the intertidal zone along the coast of the Yellow Sea, six species of common seaweeds were collected from 11 sites in 2021. The mean abundance of epiphytic nematodes in the seaweeds (e.g. *Ulvalactuca*, *Gracilariatenuistipitata*, and *Sargassumthunbergii*) was 3502 ind./g algae (dry weight). Forty species belonging to 29 genera, 16 families, and seven orders were identified. The main species were *Neochromadorapoecilosomoides* (Filipjev, 1918) Micoletzky, 1924, *Chromadorinagermanica* (Bütschli, 1874) Wieser, 1954, *Oncholaimussinensis* Zhang & Platt, 1983, and *Thalassomonhysterasiamensis* Kito & Aryuthaka, 1998. Among these materials collected was an unknown species, which is identified as new to science; it is described here as *Halomonhysterazhangi* sp. nov.

The genus *Halomonhystera* was proposed by Andrássy (2006) to accommodate marine species previously included within the genus *Geomonhystera*. Tchesunov et al. (2015) reviewed the genus and gave an annotated list of 21 morphospecies. According to NeMys database (Nemys Eds 2024), 19 *Halomonhystera* species are accepted as valid. *Halomonhysteraambiguoides* (Bütschli, 1874) is considered a *taxon inquirenda*. *Halomonhysteraparadisjuncta* (De Coninck, 1943) is accepted as *H.disjuncta* (Bastian, 1865) Andrássy, 2006. *Halomonhysterazhangi* sp. nov. is the first species of the genus found in the Yellow Sea of China.

To research the diversity of free-living marine nematodes in the northern South China Sea, sediment samples were taken at many sites in the intertidal zone. Results showed that the average abundance of free-living nematodes were 1596 ind·10 cm^−2^ in the study area. The most dominant genera were *Daptonema*, *Theristus*, and *Oncholaimus*. Among them, an unrecorded species belonging to family Xyalidae was discovered, and it is described here as *Stylotheristusflagellicaudatus* sp. nov. At present, more than 300 nematode species have been identified in a study of the biodiversity of free-living marine nematodes in the South China Sea (Huang et al. 2021; Lu et al. 2022).

The genus *Stylotheristus* was established by Lorenzen in 1977 and, until now, included only two species, namely *S.mutilus* (Lorenzen, 1973) Lorenzen, 1977 and *S.multipapillatus* Pinto & Neres, 2020. *Stylotheristusflagellicaudatus* sp. nov. is also the first species recorded within the genus in the South China Sea.

## ﻿Materials and methods

Samples of *Sargassumthunbergii* (seaweed) containing specimens of *Halomonhystera* were collected using a shovel from the intertidal rocky reef of Qingdao along the Yellow China sea (36°37.43′N, 120°18.9′E) in May 2021. The whole algal samples were scooped off at the roots, then fixed with equivalent 10% formalin in seawater for long-term preservation. In the laboratory, algal samples were poured into a beaker with filtered water, shaken, and washed. Then washing liquid was poured into two layers of sieves (500 and 42 µm mesh sizes, respectively), and washed with tap water to remove silt and to separate macrofauna from meiofauna. Heavier sediment particles were removed using centrifugation in Ludox-TM (50% colloidal silica, suspension in water; product of Sigma Aldrich Co., USA) with a specific gravity of 1.15 g/ml (de Jonge and Bouwman 1977). Each sample was washed into a Petri dish with distilled water, and the meiofauna was sorted under a stereoscopic microscope. Nematodes were transferred into a cavity block containing a solution of 5% glycerol, 5% pure ethanol, and 90% freshwater by volume (McIntyre and Warwick 1984). After ethanol was slowly evaporated, the specimens were mounted in glycerin on permanent slides. The descriptions were made using a differential interference contrast microscope (Leica DM 2500). Line drawings were made with the aid of a camera lucida. All measurements were obtained using Leica LAS X v. 3.3.3, and all curved structures were measured along the arc or median line.

Sediment samples containing specimens of *Stylotheristus* were collected at an intertidal muddy beach of Sangengzhi along Hainan Island in the South China Sea (19°26′55″N, 108°37′38″E) in March 2017. The samples were taken from the 0–8 cm sediment layer using a 2.9 cm diameter sawn-off syringe, then fixed with 10% formalin in filtered seawater for long-term preservation. In the laboratory, the samples were stained with 0.1% rose Bengal, poured into two layers of sieves (500 and 42 µm mesh sizes), and washed with tap water to remove silt and separate macrofauna from meiofauna. The following experimental procedure was as mentioned above.

Abbreviations are as follows: a, the ratio of body length to maximum body diameter; abd, body diameter at cloaca or anus; b, ratio of body length to pharynx length; c, ratio of body length to tail length; cbd, corresponding body diameter; c′, ratio of tail length to cloacal or anus body diameter; V%, position of vulva from anterior end expressed as a percentage of total body length.

## ﻿Results and discussion

### ﻿Taxonomy

**Order Monhysterida Filipjev, 1929**

**Family Monhysteridae de Man, 1876**

#### ﻿Genus Halomonhystera

Taxon classificationAnimaliaMonhysteridaMonhysteridae

Andrássy, 2006

57C9B984-A7E3-550B-AFE4-4E60FF973C9A

##### Diagnosis.

Cuticle thin, smooth with few somatic setae; labial region not or only slightly off-set; outer labial and cephalic setae short, usually no longer than one-quarter of labial width; buccal cavity cuticularized, cup-shaped or funnel-shaped; amphidial fovea circular, located one to three head diameters from anterior end; pharynx relatively short, without basal bulb; secretory–excretory system well developed with large ventral gland and opening in anterior third of pharynx; females with single anterior ovary to the right of intestine; vulva usually close to anus; males with single outstretched testis; spicules thin, arcuate, with or without a capitulum; gubernaculum short, often with caudal apophysis; one or two ventral precloacal papillae and two or three pairs of smaller caudal papillae; rectum short and thin; tail conical, usually shorter in males than females; three or two caudal glands; spinneret surrounded by tube-like structure (modified from Leduc 2014).

#### ﻿ Halomonhystera zhangi sp. nov.

Taxon classificationAnimaliaMonhysteridaMonhysteridae

AD50AEDC-26D5-5CCA-8254-186D0D8C4E5E

https://zoobank.org/F20E976D-2B9F-48D2-9C8F-58E0656741F2

**Figure 1.**
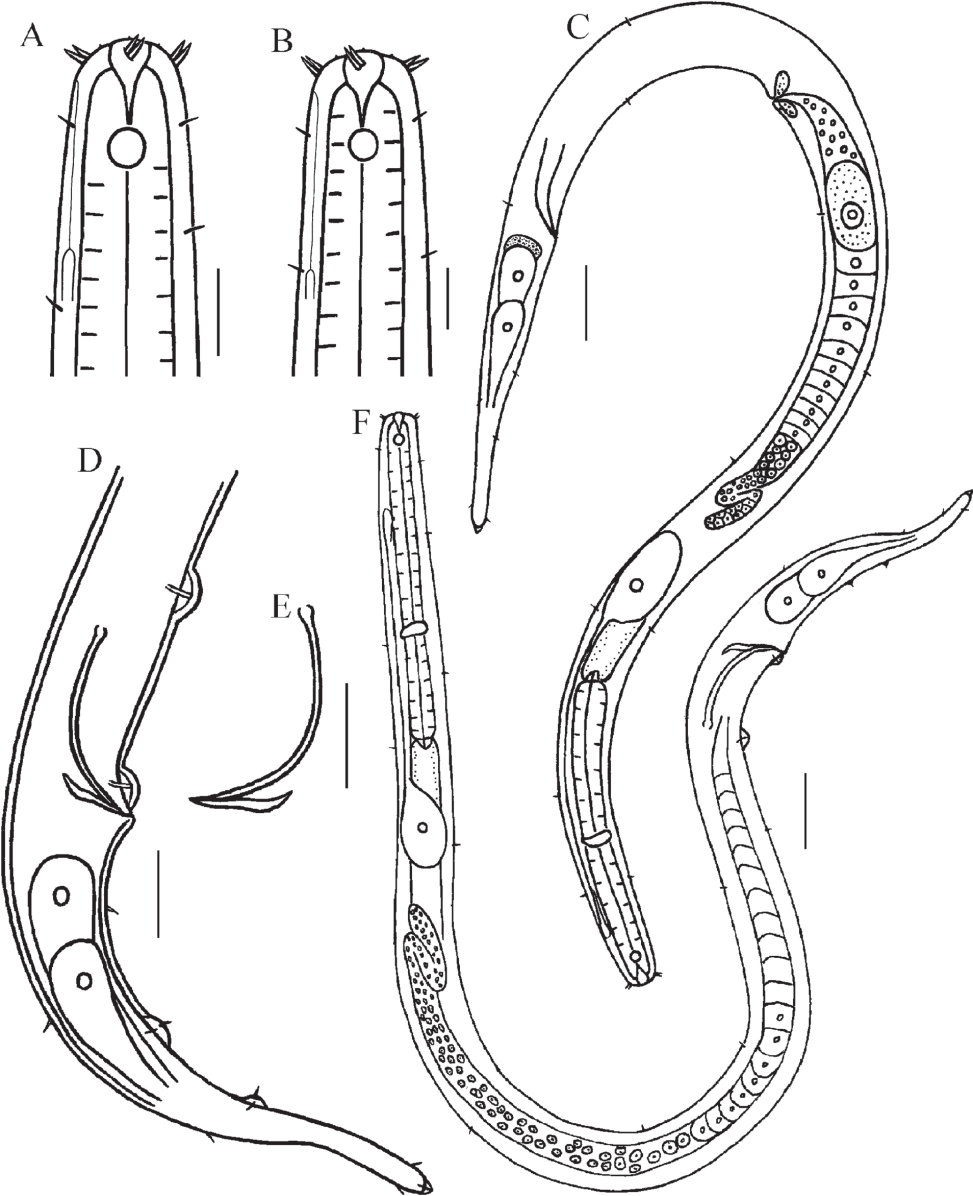
Drawings of *Halomonhysterazhangi* sp. nov. **A** anterior end of male **B** anterior end of female **C** entire body of female **D** posterior end of male **E** spicules and gubernaculum **F** entire body of male. Scale bars:10 μm (**A, B**); 30 μm (**C, F**); 20 μm (**D, E**).

**Figure 2.**
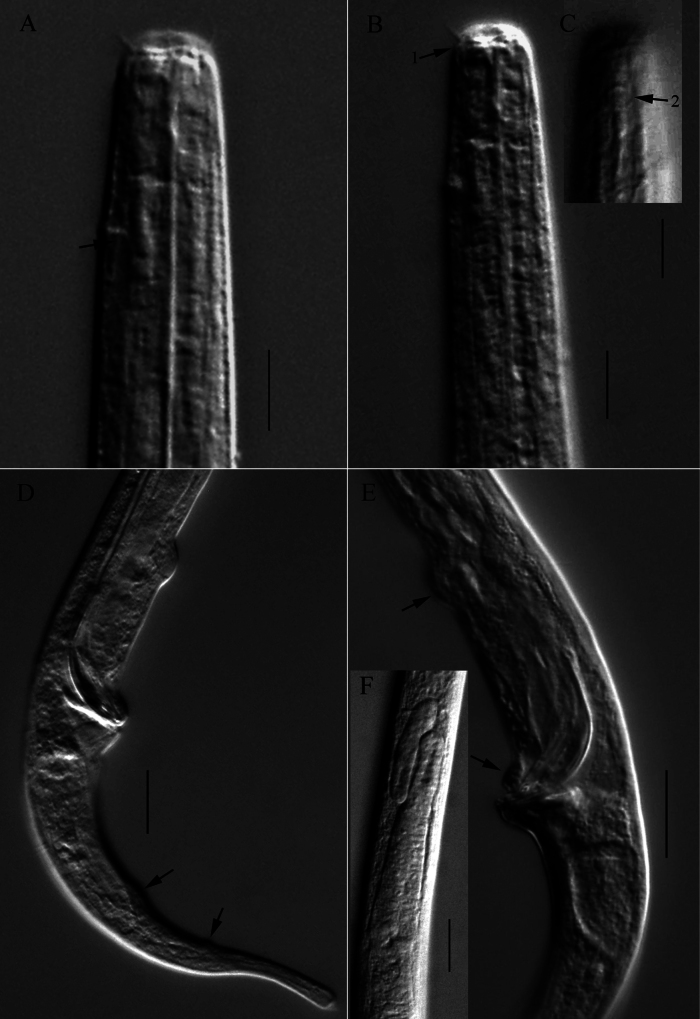
Microscopic images of *Halomonhysterazhangi* sp. nov. **A** anterior end of holotype, showing anterior setae, renette ampulla (arrow) **B, C** anterior end of holotype, showing buccal cavity, cephalic setae, excretory pore (arrow 1) and amphidial fovea (arrow 2) **D** posterior end of holotype, showing gubernaculum and caudal papillae (arrows) **E** cloacal region of paratype 1, showing spicule and precloacal papillae (arrows) **F** anterior portion of testis of holotype. Scale bars: 10 μm (**A**–**E**); 20 μm (**F**).

**Figure 3.**
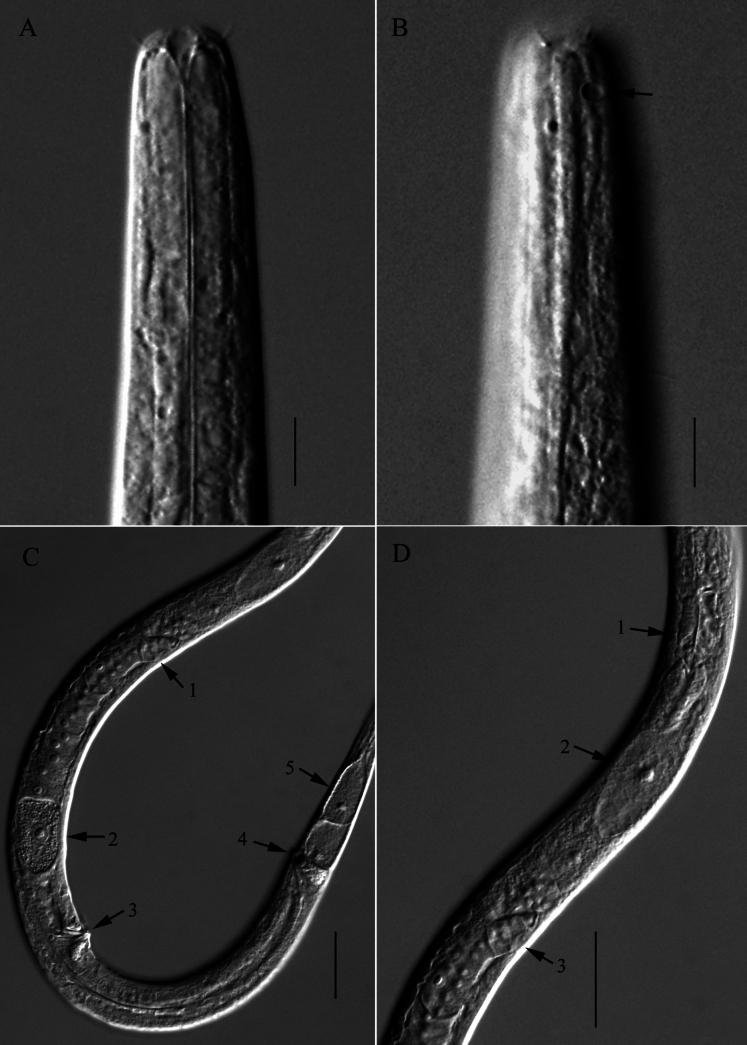
Microscopic images of *Halomonhysterazhangi* sp. nov. **A** anterior end of female 1, showing buccal cavity and anterior setae **B** anterior end of female 2, showing cephalic setae and amphidial fovea (arrow) **C** middle region of female, showing ovary (arrow 1), egg (arrow 2), vulva (arrow 3), anus (arrow 4) and caudal glands (arrow 5) **D** anterior region of female intestine, showing pharyngeal base (arrow 1), ventral gland (arrow 2) and ovary (arrow 3). Scale bars: 10 μm (**A, B**); 30 μm (**C, D**).

Figs 1 , 2 , 3 , Table 1

##### Diagnosis.

*Halomonhysterazhangi* sp. nov. is characterized by relatively small body size, anterior sensory setae 3 µm long; small circular amphidial fovea located at the level of buccal cavity base; buccal cavity funnel-shaped; excretory-secretory system with large ventral gland and opening close to the level of cephalic setae by a very thin canal; slender spicules curved slightly with cephalated proximal end and tapered distal end; gubernaculum rod-like, without apophysis; two papilliform precloacal supplements just in front of cloaca; tail conico-cylindrical with two ventral papillae and each with a seta; testis outstretched with folded anterior portion, situated at the right side of the intestine; distance between the vulva and anus longer than the tail length.

##### Material examined.

Four males and two females were obtained. ***Holotype***: ♂#1 on slide QDZQ-16JL-89; ***paratypes***: ♂#2 on slide QDZQ-11SC-148, ♂#3 on slide QDZQ-11SC-157, ♂#4 on slide QDZQ-16JL-93, ♀#1 on slide QDZQ-16JL-97, and ♀#2 on slide QDZQ-16JL-98. Type specimens were deposited in the Marine Biological Museum of the Chinese Academy of Sciences, Qingdao.

##### Type locality and habitat.

Holotype and all the additional specimens were found from *Sargassumthunbergii* (seaweed) growing on the intertidal rocky reef of Qingdao Trestle, China (36°37.43′N, 120°18.9′E).

##### Etymology.

The specific epithet “zhangi” is in honor of Professor Zhinan Zhang, a Chinese nematologist, in recognition of his contributions to nematode taxonomy.

##### Measurements.

All measurement data are given in Table 1.

**Table 1.** Measurements of *Halomonhysterazhangi* sp. nov. (in µm except for ratios).

Characters	Holotype	Paratypes	
	male	males (*n* = 3)	females (*n* = 2)
Total body length	820	732.3 ± 68.5 (654–781)	677.0 ± 67.9 (629–725)
Maximum body diameter	28	25.3 ± 1.2 (24–26)	27.0 ± 2.8 (25–29)
Head diameter	11	10.7 ± 0.6 (10–11)	13.5 ± 0.7 (13–14)
Length of cephalic setae	3	3.0 ± 0.0 (3)	4.3 ± 0.4 (4–4.5)
Depth of buccal cavity	7	6.3 ± 0.6 (6–7)	6.0 ± 0 (6–6)
Width of buccal cavity	4	4.3 ± 0.6 (4–5)	3.5 ± 0.7 (3–4)
Amphidial fovea diameter	4	4.0 ± 0.0 (4)	3.0 ± 0 (3–3)
Amphidial fovea from anterior end	7	7.7 ± 0.6 (7–8)	7.0 ± 0 (7–7)
Body diameter at amphidial fovea level	14	13.3 ± 0.6 (13–14)	14.5 ± 0.7 (14–15)
Nerve ring from anterior end	89	81.7 ± 5.5 (78–88)	68.5 ± 9.2 (62–75)
Body diameter at nerve ring level	22	20.0 ± 1.0 (19–21)	22.0 ± 1.4 (21–23)
Pharynx length	134	128.7 ± 5.5 (123–134)	116.5 ± 12.0 (108–125)
Body diameter at base of pharynx	23	21.3 ± 1.5 (20–23)	23.0 ± 1.4 (22–24)
Spicules length along arc	47	43.3 ± 2.1 (41–45)	–
Length of gubernaculum	20	17.0 ± 1.0 (16–18)	–
Body diameter at cloaca or anus	29	26.0 ± 2.6 (23–28)	22.0 ± 2.8 (20–24)
Tail length	136	118.3 ± 14.6 (102–130)	114.5 ± 10.6 (107–122)
Vulva from anterior end	–	–	393.5 ± 37.5 (367–420)
Body diameter at vulva	–	–	28.5 ± 3.5 (26–31)
V%	–	–	58.1 ± 0.3 (57.9–58.3)
a	29.3	28.9 ± 1.4 (27.3–30.0)	25.1 ± 0.1 (25.0–25.2)
b	6.1	5.7 ± 0.6 (5.3–6.4)	5.8 ± 0 (5.8–5.8)
c	6.0	6.2 ± 0.2 (6.0–6.4)	5.9 ± 0 (5.9–5.9)
c′	4.7	4.5 ± 0.2 (4.4–4.8)	5.3 ± 0.2 (5.1–5.4)

##### Description.

**Males.** Body slender, tapering towards both extremities. Cuticle smooth without transversely striated. Four longitudinal rows of short somatic setae sparsely distributed throughout the body, 3–4 µm long. Head diameter representing 39–42% of the maximum body diameter. Inner labial sensilla papilliform. Outer labial sensilla setiform. Outer labial setae and cephalic setae united in one circle with a total of 12 setae, each 3 µm long, i.e. 27–30% of head diameter, situated at the level of middle of the buccal cavity. Amphidial fovea circular with a diameter of 4 µm, which is occupying 29–31% of the corresponding body diameter, located at the level of buccal cavity base, i.e.7–8 µm from the anterior end. Buccal cavity funnel-shaped, without teeth. Pharynx cylindrical with a swollen base, not forming a real terminal bulb. Cardia conical, 6–9 µm long. Nerve ring situated posterior to the middle of pharynx. Excretory–secretory system with a large ventral cell, situated near anterior section of intestine; ampulla situated posterior to amphidial fovea, about 25 µm from the anterior end of body; a very thin canal extended forwards from the ampulla, and opening just posterior to the level of outer labial and cephalic setae crown. Tail conical with posterior third cylindrical, equal to 4.4–4.8 cloacal body diameter long. Tail tip slightly swollen with a conical hyaline spinneret (Fig. 2D). Terminal setae absent. A few short caudal setae sparsely distributed throughout the tail. Two prominent caudal gland cells confined entirely to the tail.

Reproductive system monorchic with an outstretched testis with folded anterior portion, situated at the right side of the intestine. (Fig. 2F). Spicules slender, arcuated, proximally cephalated and distally pointed, 1.52–1.96 times of cloacal body diameter. Gubernaculum rod-like, slightly curved proximally, without apophysis. Two papilliform precloacal supplements, one just anterior to cloaca, another one 30–50 µm anterior to cloaca. Two ventral caudal papillae in the middle region of tail, each with a short seta. The anterior one located at 43–52 µm posterior from cloaca, and the other at 63–72 µm posterior from cloaca.

**Females.** Similar to males in most morphological characteristics except cephalic setae slightly longer and amphidial fovea relatively smaller. Reproductive system monodelphic with an anterior outstretched ovary, located at the right side of intestine. Mature egg large, oval. Uterus a wide tube. Vulva located posterior to mid-body (i.e. 57.9–58.3% of body length from the anterior end). Distance between the vulva and anus (155–183 µm) longer than the tail length (107–122 µm). Spermatheca not seen.

##### Differential diagnosis and discussion.

*Halomonhysterazhangi* sp. nov. agrees well with the primary diagnostic characters of the genus, especially in having well developed excretory–secretory system with large ventral gland and opening at anterior pharyngeal region, spinneret with hyaline structure, males having papillary precloacal supplements and caudal papillae. The inconsistent characters to diagnosis of *Halomonhystera* are cephalic setae paired and vulva not very close to anus.

The present species is similar to *H.chitwoodi* (Steiner, 1958) Andrássy, 2006 in the position of amphidial fovea (closing to anterior end of body), the distance between vulva and anus (not shorter than the tail length) and they all growing on Sargassum, but it differs from the latter species by slightly smaller body size (vs longer than 1 mm), two papilliform precloacal supplements (vs only one precloacal supplement), and conico-cylindrical tail with two ventral bristled papillae (vs conical tail without bristled papilla). In having relatively long distance between the vulva and anus, the new species resembles *H.glaciei* (Blome & Riemann, 1999) Andrássy, 2006, but differs from it by the much shorter and stouter body (vs 2 mm or more, a = 60 or more), the amphidial fovea closing to the anterior body end (vs two labial diameters from anterior end) and by the shorter tail (vs c′= 7). The new species can easily be distinguished from other known species within this genus by relatively small body size, position of amphidial fovea near to anterior end of body, two papilliform precloacal supplements, conico-cylindrical tail with two ventral bristled papillae. The difference between *H.zhangi* sp. nov. and other known species within the genus can be inferred from the key below.

### ﻿Updated key to all species of *Halomonhystera* (based on Leduc 2014; Tchesunov et al. 2015)

**Table:** 

1	Distance from vulva to anus 4–12 abd, equal or longer than tail length	**2**
–	Distance from vulva to anus 1–3 abd, much shorter than tail length	**8**
2	Body longer than 2 mm, extremely slender (a = 60–91)	***H.glaciei* (Blome & Riemann, 1999)**
–	Body shorter than 1.5 mm, moderately slender (a = 20–38)	**3**
3	Body very short, 357–400 µm, spicules 14–19 µm	***H.islandica* (De Coninck, 1943)**
–	Body longer than 600 µm, spicules longer than 22 µm	**4**
4	Spicules longer than 40 µm	**5**
–	Spicules equal or shorter than 30 µm	**6**
5	Tail conico-cylindrical with two ventral bristled papillae	***H.zhangi* sp. nov.**
–	Tail conical without bristled papilla	***H.chitwoodi* (Steiner, 1958)**
6	Tail elongated, both males and females longer than 5.5 abd, spicules 22 µm long	***H.bathislandica* (Riemann, 1995)**
–	Tail length medium, spicules 23–30 µm long	**7**
7	Width of amphidial fovea equal to 50% cbd, females ovoviviparous	***H.fisheri* (Zekely, Sorensen & Bright, 2006)**
–	Width of amphidial fovea equal to 40% cbd, females oviparous	***H.vandoverae* (Zekely, Sorensen & Bright, 2006)**
8	Body longer than 1.8 mm, a = 40–50, tail with three successive pairs of subventral papillae in males, spicules 46–96 µm long	***H.socialis* (Bütschli, 1874)**
–	Body shorter than 1.6 mm, a = 20–43, spicules 23–53 µm long	**9**
9	Body small (440–460 µm long) with relatively longer tail (c′ = 6.5)	***H.uniformis* (Cobb, 1914)**
–	Body size medium, with relatively shorter tail	**10**
10	Distance from amphidial fovea to anterior body end 2–3 cbd, spicules 38 µm long	***H.cameroni* (Steiner, 1958)**
–	Distance from amphidial fovea to anterior body end less than 2 cbd	**11**
11	Width of amphidial fovea equal to 33–50% cbd	**12**
–	Width of amphidial fovea less than 32% cbd	**14**
12	Spicules 34–40 µm long	***H.antarctica* (Cobb, 1914)**
–	Spicules 25–30 µm long	**13**
13	Body plump, 420–580 µm long, a = 20–24	***H.continentalis* Andrássy, 2006**
–	Body slender, 605–780 µm long, a = 28–34	***H.hickeyi* Zekely, Sorensen & Bright, 2006**
14	Females ovoviviparous	**15**
–	Females oviparous	**17**
15	Amphid located 1.5–1.9 labial diameters from the anterior body end, gubernaculum without or with inconspicuous apophysis	***H.disjuncta* (Bastian, 1865)**
–	Amphid located more anteriorly (0.7–1.4 labial diameters), gubernaculum with conspicuous apophysis	**16**
16	Tail with three successive pairs of subventral papillae in males	***H.hermesi* Tchesunov, Portnova & van Campenhout, 2015**
–	Tail with only one pair of subventral papillae close to the tail tip in males	***H.halophila* Andrássy, 2006**
17	Distance from vulva to anus 4.2 abd, equal to 85% tail length	***H.rotundicapitata* (Filipjev, 1922)**
–	Distance from vulva to anus less than 2 abd, shorter than 35% tail length	**18**
18	Gubernaculum with caudal apophyses, tail with four pairs post-cloacal papillae in males	***H.tangaroa* Leduc, 2014**
–	Gubernaculum without caudal apophyses, tail without or with two pairs post-cloacal papillae in males	**19**
19	Buccal cavity with denticles, spicules 41–44 µm long	***H.parasitica* Poinar, Duarte & Santos Maria, 2009**
–	Buccal cavity without denticles, spicules 23–30 µm	***H.taurica* (Tsalolikhin, 2007)**

#### ﻿Family Xyalidae Chitwood, 1951

##### ﻿Genus Stylotheristus

Taxon classificationAnimaliaMonhysteridaXyalidae

Lorenzen, 1977

CC830091-309A-5DA9-9BC2-48515810A5CF

###### Diagnosis.

Anterior sensilla arranged in two crowns with the number of setae in the second crown depending on the sex and life stage, 6+4 in females and juveniles and 6+10 in males, inner labial sensilla conical; buccal cavity conical; pharyngeal muscles well-developed around the buccal cavity; amphidial fovea transversely oval; spicules short; spermatheca present on the right side of intestine; three caudal glands opening at separate pores; tail conico-cylindrical with three terminal setae (Pinto and Neres 2020).

##### ﻿ Stylotheristus flagellicaudatus sp. nov.

Taxon classificationAnimaliaMonhysteridaXyalidae

C5446C0C-ED97-59DF-8D87-6CEFA8FE88E7

https://zoobank.org/3875D927-471F-4BEF-AF20-41B8F80B3380

**Figure 4.**
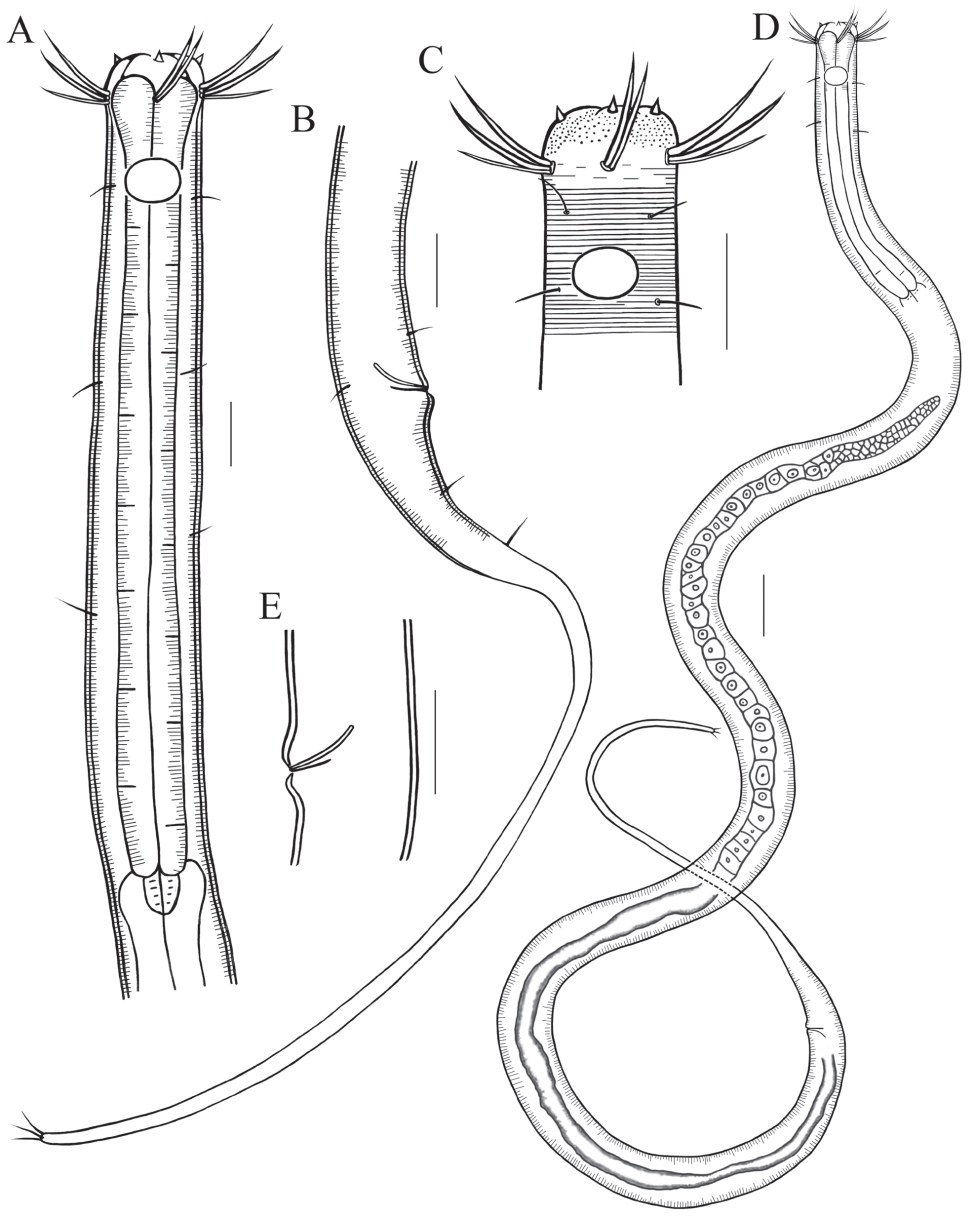
Drawings of *Stylotheristusflagellicaudatus* sp. nov. **A** lateral view of pharyngeal region of holotype **B** lateral view of posterior portion, showing long conico-cylindrical tail **C** lateral view of anterior portion of holotype, showing the two circles of anterior setaes and oval amphideal fovea **D** entire view of male **E** cloacal region of holotype, showing spicule and gubernaculum. Scale bars: 20 µm (**A**–**C, E**), 30 µm (**D**).

**Figure 5.**
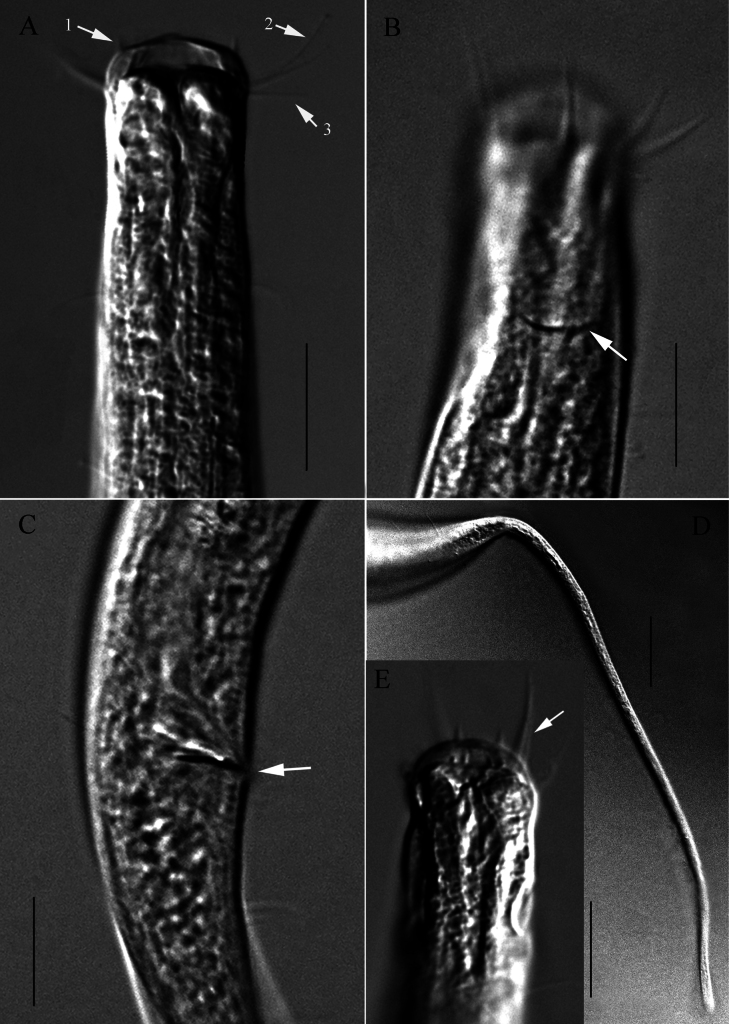
Microscopic images of *Stylotheristusflagellicaudatus* sp. nov. **A** lateral view of anterior portion of holotype, showing conical inner labial setae (arrow 1), outer labial setae (arrow 2), subcephalic setae (arrow 3) and buccal cavity **B** lateral view of anterior portion of holotype, showing cephalic setae and amphideal fovea (arrow) **C** lateral view of cloacal region of holotype, showing spicule and gubernaculum (arrow) **D** lateral view of posterior portion of paratype 1, showing filiform tail and caudal setae **E** dorsal view of anterior portion of paratype 3, showing cephalic setae (arrow). Scale bars: 20 µm.

Figs 4 , 5 , Table 2

###### Diagnosis.

*Stylotheristusflagellicaudatus* sp. nov. is characterized by relatively shorter body and longer tail than that of two species already described in this genus; cuticle striated; anterior sensilla arranged in two circles: the first circle consisting of six inner labial setiform papillae (3–4 µm), the second circle consisting of 16 long setae (12–19 µm); amphideal fovea transversely oval; well-developed muscle around funnel-shaped buccal cavity; spicules short, gubernaculum composed of a single piece, precloacal supplements absent; tail elongated, filiform.

###### Type material.

Four males were collected. ***Holotype***: ♂#1 on slide Sangengzhi 87-4. ***Paratypes***: ♂#2 on slide Sangengzhi 81-10, ♂#3 on slide Sangengzhi 81-11 and ♂#4 on slide Sangengzhi 37-3.

###### Type locality and habitat.

Holotype and other specimens were collected in the muddy sediment from the intertidal zone of Sangengzhi, Hainan Province (19°26′55″N, 108°37′38″E).

###### Etymology.

The species epithet “flagellicaudatus” refers to its long and filiform tail.

###### Measurements.

Measurements are given in Table 2.

**Table 2.** Measurements of the *Stylotheristusflagellicaudatus* sp. nov. (in µm except for ratios).

Characters	Holotype	Paratypes
	male	males (*n* = 3)
Total body length	1683	1570.7 ± 39.3 (1526–1600)
Maximum body diameter	30	31.7 ± 2.1 (31–34)
Head diameter	23	23.7 ± 1.2 (23–25)
Length of inner labial setae	4	3.0 ± 0 (3)
Length of outer labial setae	18	19.3 ± 1.2 (18–20)
Length of cephalic setae	18	19.3 ± 1.2 (18–20)
Amphideal fovea as percentage of corresponding body diameter	45	45.0 ± 5.0 (40–50)
Amphideal fovea from anterior end	24	25.0 ± 0 (25)
Pharynx length	201	191.7 ± 5.7 (187–198)
Body diameter at pharyngeal base	36	29.3 ± 1.2 (28–30)
Length of spicules	15	14.0 ± 2.6 (11–16)
Cloacal body diameter	25	24.7 ± 0.6 (24–25)
Tail length	310	299.7 ± 0.6 (299–300)
a	56.1	49.5 ± 4.4 (44.9–53.3)
b	8.4	8.2 ± 0.1 (8.1–8.3)
c	5.4	5.2 ± 0.2 (5.1–5.5)
c’	12.4	12.1 ± 0.3 (12.0–12.5)

###### Description.

**Males.** Body slender, cylindrical, and gradually tapering towards tail end. Cuticle striated. Six longitudinal lines of short somatic setae sparsely distributed throughout the body, 4–6 µm long. Anterior sensilla arranged in two circles: the first circle consisting of six inner labial setiform papillae, conical, 3–4 µm long; the second circle consisting of six outer labial setae (18–19 µm), four cephalic setae (16–17 µm) and six subcephalic setae (12–13 µm), situated at the level of buccal cavity base. Buccal cavity funnel-shaped, with well-developed pharyngeal muscles around it. Pharynx cylindrical, not expanded at posterior end. Amphideal fovea transversely oval, 8 µm high and 11–12 µm wide, occupying 40–50% of corresponding body diameter, located at the position of 24–25 µm from the anterior end. Nerve ring located at the middle of pharynx. Secretory–excretory pore not observed. Tail elongated, conico-cylindrical, posterior three-quarters filiform. Three terminal setae 10 µm long, three caudal glands present.

Reproductive system monorchid, an anterior testis outstretched, to the left side of intestine. Spicules short and thin, almost straight, 46–64% of cloacal body diameter long. Gubernaculum simple, short, and laminar, about 70% of spicules length. Precloacal supplements absent.

**Females** not found.

## ﻿Differential diagnosis and discussion

**Figure 6.**
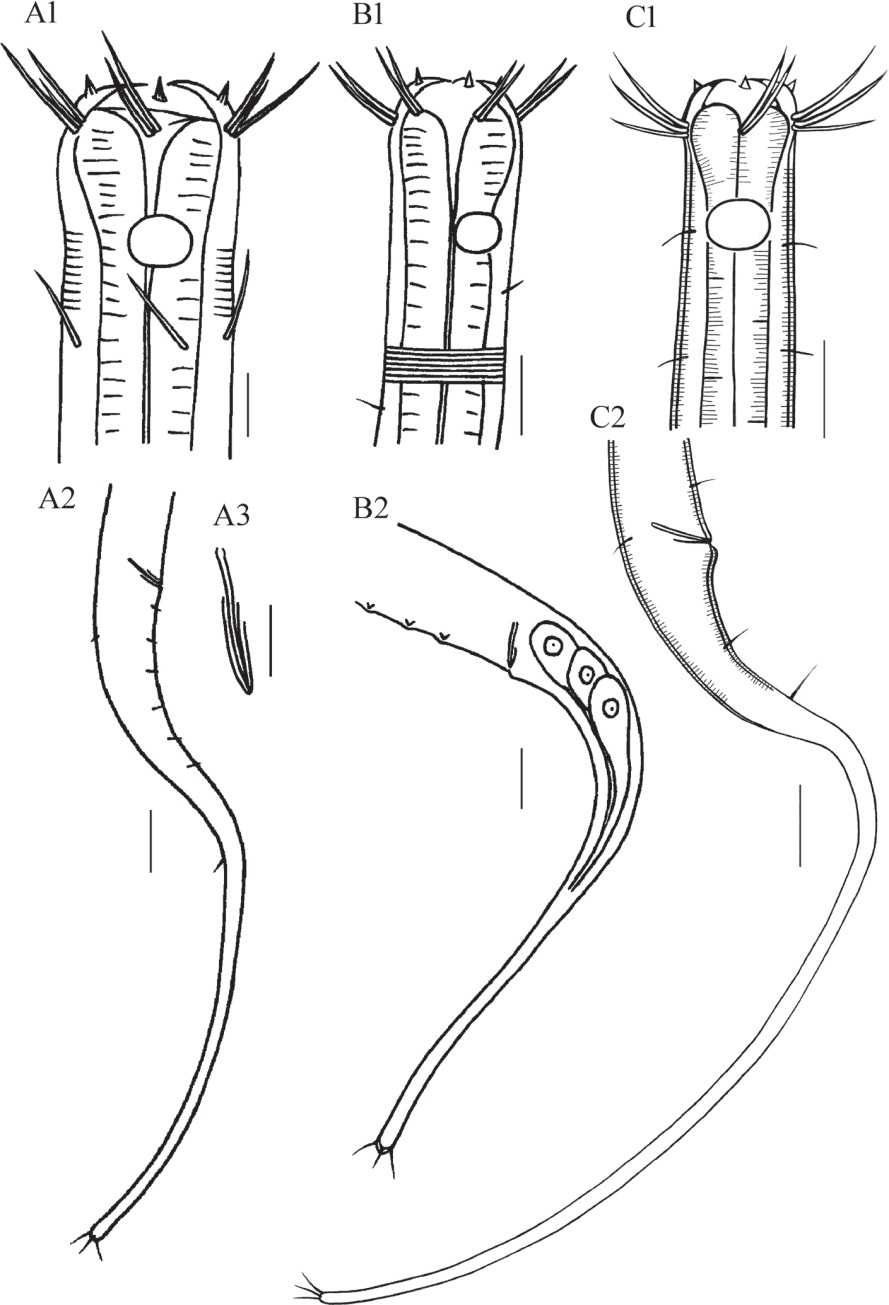
Pictorial key for genus *Stylotheristus***A***S.mutilus* (from Lorenzen 1973) **B***S.multipapillatus* (from Pinto and Neres 2020) **C***S.flagellicaudatus* sp. nov. Scale bars: 20 µm (**A1, B, C**), 50 µm (**A2**), 10 µm (**A3**).

The characteristics of the new species match well with the main diagnostic of *Stylotheristus* (Fonseca and Bezerra 2014). The genus only has contained only two species until now, i.e. *S.mutilus* (Lorenzen, 1973) Lorenzen, 1977 and *S.multipapillatus* Pinto & Neres, 2020. The new species is distinguished from *S.multipapillatus* by the absence of precloacal supplements, relatively larger amphidial fovea, and a longer filiform tail. The latter species possess 11–15 papilliform precloacal supplements and a smaller amphidial fovea (32–38% of corresponding body diameter). The new species differs from *S.mutilus* by absence of inerratic circle of long cervical setae, relatively larger amphidial fovea (vs 37% of corresponding body diameter), shorter spicules not cephalated proximally (vs 18–20 µm with cephalated proximal ends) and the different structure of gubernaculum (only a piece). In *S.mutilus*, the gubernaculum is formed by two pieces, it has a circle of long cervical setae. Besides that, the body size of new species is smaller than of the two known species but the tail in the new species is relatively longer (Fig. 6).

## Supplementary Material

XML Treatment for Halomonhystera

XML Treatment for Halomonhystera zhangi

XML Treatment for Stylotheristus

XML Treatment for Stylotheristus flagellicaudatus

## ﻿Additional information

### Author ORCIDs

Ting Li https://orcid.org/0009-0003-5630-314X

Yong Huang https://orcid.org/0000-0002-1846-8088

Mian Huang https://orcid.org/0000-0003-3343-1520

### Data availability

All of the data that support the findings of this study are available in the main text.
